# No benefit of HIF prolyl hydroxylase inhibition for hypertensive renal damage in renovascular hypertensive rats

**DOI:** 10.3389/fphys.2023.1208105

**Published:** 2023-06-26

**Authors:** Andrea Hartner, Thomas Dambietz, Nada Cordasic, Carsten Willam, Nicolai Burzlaff, Martin Brötsch, Christoph Daniel, Mario Schiffer, Kerstin Amann, Roland Veelken, Gunnar Schley, Karl F. Hilgers

**Affiliations:** ^1^ Department of Pediatrics and Adolescent Medicine, University of Erlangen-Nürnberg, Erlangen, Germany; ^2^ Department of Nephrology and Hypertension, University of Erlangen-Nürnberg, Erlangen, Germany; ^3^ Department of Chemistry and Pharmacy, University of Erlangen-Nürnberg, Erlangen, Germany; ^4^ Department of Nephropathology, University of Erlangen-Nürnberg, Erlangen, Germany

**Keywords:** capillarization, vascular lesions, malignant hypertension, HIF stabilization, renovascular

## Abstract

**Introduction:** We previously reported that malignant hypertension is associated with impaired capillary density of target organs. Here, we tested the hypothesis that stabilization of hypoxia-inducible factor (HIF) in a modified “preconditioning” approach prevents the development of malignant hypertension. To stabilize HIF, we employed pharmacological inhibition of HIF prolyl hydroxylases (PHD), that profoundly affect HIF metabolism.

**Methods:** Two-kidney, one-clip renovascular hypertension (2K1C) was induced in rats; controls were sham operated. 2K1C rats received either intermittent injections of the PHD inhibitor ICA (2-(1-chloro-4-hydroxyisoquinoline-3-carboxamido) acetate) or placebo. Thirty-five days after clipping, the frequency of malignant hypertension was assessed (based on weight loss and the occurrence of characteristic vascular lesions). In addition, kidney injury was compared between all ICA treated versus all placebo treated 2K1C, regardless of the occurrence of malignant hypertension. HIF stabilization was evaluated by immunohistochemistry, and HIF target gene expression by RT-PCR.

**Results:** Blood pressure was elevated to the same degree in ICA- and placebo-treated 2K1C compared to control rats. ICA treatment did not affect the frequency of malignant hypertension or the extent of kidney tissue fibrosis, inflammation, or capillary density. There was a trend towards higher mortality and worse kidney function in ICA-treated 2K1C rats. ICA increased the number of HIF-1α-positive renal tubular cell nuclei and induced several HIF-1 target genes. In contrast, expression of HIF-2α protein as well as HIF-2 target genes were markedly enhanced by 2K1C hypertension, irrespective of ICA treatment.

**Discussion:** We conclude that intermittent PHD inhibition did not ameliorate severe renovascular hypertension in rats. We speculate that the unexpected strong renal accumulation of HIF-2α in renovascular hypertension, which could not be further augmented by ICA, may contribute to the lack of a benefit from PHD inhibition.

## Introduction

The development of so-called malignant hypertension, characterized by progressive target organ damage, is a serious complication of increased blood pressure (Domek et al., 2020). Typical features of this condition are progressive target organ damage, including characteristic vascular lesions resembling thrombotic microangiopathy in kidney tissue (Palma et al., 2021). It remains unclear, however, why some hypertensive patients develop a malignant course of the disease, while others do not. In a previous study, we described impaired neovascularization and reduced capillary supply in end organ tissue of the malignant course of our model of renovascular hypertension (Hartner et al., 2016). In this model, a silver clip is placed on the left renal artery of young rats; as the animals grow, the clip starts to constrict the renal artery, leading to renin release and the development of hypertension (Hilgers et al., 2001). About 2 weeks after clipping, some of these rats start to develop malignant hypertension, heralded clinically by weight loss and hypovolemia. Progressive target organ damage accompanied by capillary rarefaction and characteristic lesions of arterioles then occur. These vascular peculiarities were not detected in benign hypertension in our model. Therefore, we hypothesized that reduced vascularization might contribute to the development of end organ damage and poor outcome. Stimulation of angiogenesis seemed to be a promising tool to ameliorate the course of hypertensive nephropathy. Several studies have shown that angiogenesis can be induced or improved by stabilization of hypoxia-inducible transcription factors (HIFs). HIFs are the major regulators of the cellular responses to hypoxia and activate the expression of a variety of target genes to maintain cellular oxygen homeostasis. HIFs are tightly controlled by HIF prolyl hydroxylases (PHD) enzymes, which serve as cellular oxygen sensors and regulate the oxygen-dependent degradation of HIFα subunits in the proteasome (Kremer et al., 2019; Qian et al., 2019). Besides oxygen, PHDs also require 2-oxoglutarate as a cosubstrate. Analogues of 2-oxoglutarate inhibit PHD activity mimicing hypoxia and activating many of HIF transcriptional responses under normoxic conditions. They have been demonstrated to be protective in ischemic and inflammatory diseases in various organs (Sen Banerjee et al., 2012; Hasegawa et al., 2018; Requena-Ibanez et al., 2022). The PHD inhibitor 2-(1-chloro-4- hydroxyisoquinoline-3-carboxamido) acetate (ICA) ameliorated renal injury in acute and chronic kidney disease models. ICA stabilized both HIF isoforms and induced HIF target genes, like HO-1 as well as VEGF and VEGF receptors, which are important mediators for vessel formation (Schellinger et al., 2017). In healthy mice, a single intraperitoneal injection of ICA stabilized HIF-1a for longer than 12 h and upregulated HIF target genes for more than 24 h (Schley et al., 2012). A regime of 2 intermittent doses of ICA was effective in ameliorating defective angiogenesis in experimental chronic kidney disease (Schellinger et al., 2017). Therefore, we used a similar regimen to stabilize HIFs in the 2-kidney, 1-clip (2K1C) model of renovascular hypertension. Permanent application of ICA and/or permanent stabilization of HIF over an extended time period was purposely avoided to minimize effects on hematocrit and blood pressure. We hypothesized that ICA-mediated HIF-stabilization might prevent occurrence of malignant hypertension via stimulation of angiogenesis.

## Materials and methods

### Renovascular hypertension

Rats were kept at 22°C ± 2°C, exposed to a 12 h dark/light cycle. The animals received standard chow (#1320, Altromin, Lage, Germany) and tap water *ad libitum*. All procedures performed on animals were in compliance with the DIRECTIVE 2010/63/EU of the European Parliament and were approved by the local government authorities (Regierung of Mittelfranken, AZ 54-2532.1-51/12). All experimental protocols were approved by the local government authorities. All methods are carried out in accordance with relevant guidelines and regulations. The study is reported in accordance with ARRIVE guidelines and all possible steps were taken to avoid animals’ suffering at each stage of the experiment. Two-kidney, one-clip renovascular hypertension (2K1C) was induced in male Sprague-Dawley rats (from Charles River, Sulzfeld, Germany) weighing 150–170 g by placing a silver clip of 0.2 mm internal diameter around the left renal artery through a flank incision under isoflurane anesthesia as previously described (Mai et al., 1995) (n = 39). Control animals underwent sham operation without placement of the clip (n = 9). Analgesia with subcutaneous buprenorphine injections (0.05 mg/kg) was provided routinely and as needed. Body weights and systolic blood pressure (tail cuff plethysmography) were measured repeatedly (Supplementary Figure S1). Five weeks after clipping of the renal artery, the experiment was terminated. Methods describing induction of hypertension were given in detail in (Hartner et al., 2016). A time line of the experimental procedures is given in Supplementary Figure S1.

### HIF-stabilization via pharmacological inhibition of HIF prolyl hydroxylases

The small molecule 2-(1-chloro-4-hydroxyisoquinoline-3-carboxamido) acetate (ICA) was obtained in a six-step synthesis, as previously described (Schley et al., 2012) by the coauthors N.B. and M.B. Twenty-two rats received 4 i.p. injections of ICA (1-chloro-4- hydroxyisoquinoline- 3-carboxamido acetate, dissolved in 10% dimethyl sulfoxide (DMSO), 0.5M TRIS, pH 7.4, 12.5 mg/kg body weight) as described (Schellinger et al., 2017). ICA was administered during the third week after induction of hypertension (at days 15, 16, 18, and 19 after 2K1C). Control 2K1C rats received solvent only. Another n = 10 2K1C ICA-treated and n = 10 2K1C solvent-treated rats were sacrificed at day 19 after 2K1C (4 h after ICA treatment) as a positive control to verify HIF stabilization due to ICA treatment.

### Blood pressure measurements

Systolic blood pressure was measured repeatedly by tail cuff plethysmography (Hartner et al., 2016) under light isoflurane anesthesia. At the end of the experiment, rats were instrumented with femoral artery catheters for intraarterial blood pressure measurements (Hartner et al., 2016). Measurements were performed on the same day after termination of anesthesia and a recovery phase of 2 h in conscious animals via transducers connected to a polygraph (Hellige, Freiburg, Germany). Telemetric blood pressure measurements were performed in selected animals (n = 5 in the 2K1C-placebo group and n = 5 in the 2K1C-ICA group) to monitor the blood pressure development using an implantable telemetry device (DSI, St. Paul, MN, United States). Catheters were implanted into the abdominal aorta under isoflurane anesthesia and a small transducer was placed subcutaneously. The telemetry device (Dataquest ART system, DSI) placed under the animal cage recorded the continuous blood pressure measurements between day 15 and 35 after induction of hypertension. Mean arterial blood pressure and heart rate were calculated by the Dataquest software.

### Measurement of plasma creatinine, urea, erythropoietin and aldosterone

Blood for analysis was collected from indwelling catheters. Thereafter, rats were killed by bleeding in deep anesthesia. Hematocrit was assessed using a Sigma Hematocrit centrifuge (10014, Sigma, Osterode, Germany). Plasma creatinine and urea were analysed using an automatic analyser Integra 1,000 (Roche Diagnostics, Mannheim, Germany). Plasma aldosterone was measured with a commercially available radioimmunoassay kit (Aldosterone Maia 12254, Serono Diagnostics, Freiburg, Germany). Serum erythropoietin was measured using a commercially available ELISA to rat erythropoietin (Legend Max, BioLegend, Biozol, Eching, Germany).

### Metabolic cage studies

Two days before sacrifice, rats were put in metabolic cages for 24 h to collect their urine. Albumin excretion was assessed using a commercially available ELISA (Bethyl Laboratories, Biomol, Hamburg, Germany). Albuminuria was expressed as albumin excretion per gram creatinine.

### Tissue sampling and histological analysis

After being weighed, the kidneys were decapsulated. Both poles of each kidney and the apical tip of the left ventricle were immediately snap frozen in liquid nitrogen for RNA extraction. One 6 mm slice of the kidneys and left ventricular tissue was fixed in methyl-Carnoy solution (60% methanol, 30% chloroform and 10% glacial acetic acid). Another slice was fixed in 3% paraformaldehyde. After fixation, tissue was embedded in paraffin. Paraffin-embedded tissue was sectioned and stained with periodic acid Schiff’s (PAS) reagent. Fibrinoid necroses and onion skin lesions were counted by a blinded observer in PAS-stained kidney sections of all kidneys exposed to high blood pressure (contralateral, non-clipped kidney). The criteria used for the definition of malignant hypertension were weight loss and the characteristic vascular lesions as described before (Hartner et al., 2016). Split-half analyses were performed for both criteria: weight loss and number of characteristic vascular lesions (onion skin lesions and fibrinoid necroses). Hypertension was considered as malignant if a rat was in the upper 50% for both criteria or as non-malignant if the animal was in the lower 50% for both criteria. Quantification of glomerulosclerosis was performed as described before (Menendez-Castro et al., 2018) using a semi-quantitative scoring scheme 0-4 (Supplementary Figure S2).

### Immunohistochemistry

Tissue was processed as described (Menendez-Castro et al., 2013). Renal tissue was fixed in methyl-Carnoy or paraformaldehyde and 2 µm sections were cut. Immunohistochemical detection of aminopeptidase P (JG12, BMS1104, ThermoScientific, Dreieich, Germany) was used to label microvessels of the kidney. HIF-1α was stained using a polyclonal antibody to HIF-1α (#10006421, Cayman Chemicals, Ann Arbor, MI, United States). The antibody to HIF-2α was a gift from Dr. Michael Wiesener (Wiesener et al., 2003). As secondary antibody a biotinylated goat anti-rabbit antibody (BA1000, Vector Laboratories, Biozol, Eching, Germany) or a biotinylated goat anti-mouse (BA9200, Vector Laboratories) were used. Staining was visualized using streptavidin-peroxidase (SA5004, Vector Laboratories). The staining for HIF-1α and HIF-2α was described in detail before (Schley et al., 2019). Cortical tubulointerstitial microvessels were counted in one median cross section of the right kidney. The number was related to the number of glomeruli. HIF-1α-positive nuclei were counted in 10 medium-power views of medullary and cortical kidney tissue. HIF-2α-positive nuclei were counted in 30 glomeruli of a renal section and are given as means ± error of the mean. All histological evaluations were done by a single investigator blinded to the group assignment.

### Real-time polymerase chain reaction (PCR) analyses

Myocardial and kidney tissue was homogenized in RLT buffer reagent (Qiagen, Hilden, Germany) with an ultraturrax for 30 s, total RNA was extracted from homogenates by RNeasy Mini columns (Qiagen) according to the manufacturer’s protocol and real-time RT-PCR was performed. First-strand cDNA was synthesized with TaqMan reverse transcription reagents (Applied Biosystems, Darmstadt, Germany) using random hexamers as primers. Reactions without Multiscribe reverse transcriptase were used as negative controls for genomic DNA contamination. PCR was performed with an ABI PRISM 7000 Sequence Detector System and SYBR Green Universal PCR Master Mix (Applied Biosystems). All samples were run in triplicates. Specific mRNA levels in hypertensive animals relative to sham operated controls were calculated and normalized to a housekeeping gene (18S) with the ∆-∆-C_T_ method as specified by the manufacturer (https://assets.thermofisher.com/TFS-Assets/LSG/manuals/cms_040980.pdf). The method of PCR analysis has been described before in (Hartner et al., 2016). Primer pairs used for experiments are shown in Supplementary Table S1.

### Statistical analysis

Survival was estimated using a log rank test and visualized with Kaplan-Meier curve. The relative frequency of a malignant course was cross-tabulated according to treatment and analyzed by chi^2^ test. In addition, all parameters were also analyzed as continuous variables to avoid the necessity of categorical definitions. One-way analysis of variance, followed by the LSD (Fisher’s Least Significant Differences) post-hoc test, was performed to test significance of differences between groups. Values are given as means ± error of the mean. A *p*-value <0.05 was considered statistically significant. Calculations were carried out using the SPSS 19 software (IBM, Ehningen, Germany).

## Results

### ICA treatment enhanced renal tubular HIF-1α stabilization, while glomerular HIF-2α was mainly stabilized by renovascular hypertension

**FIGURE 1:**
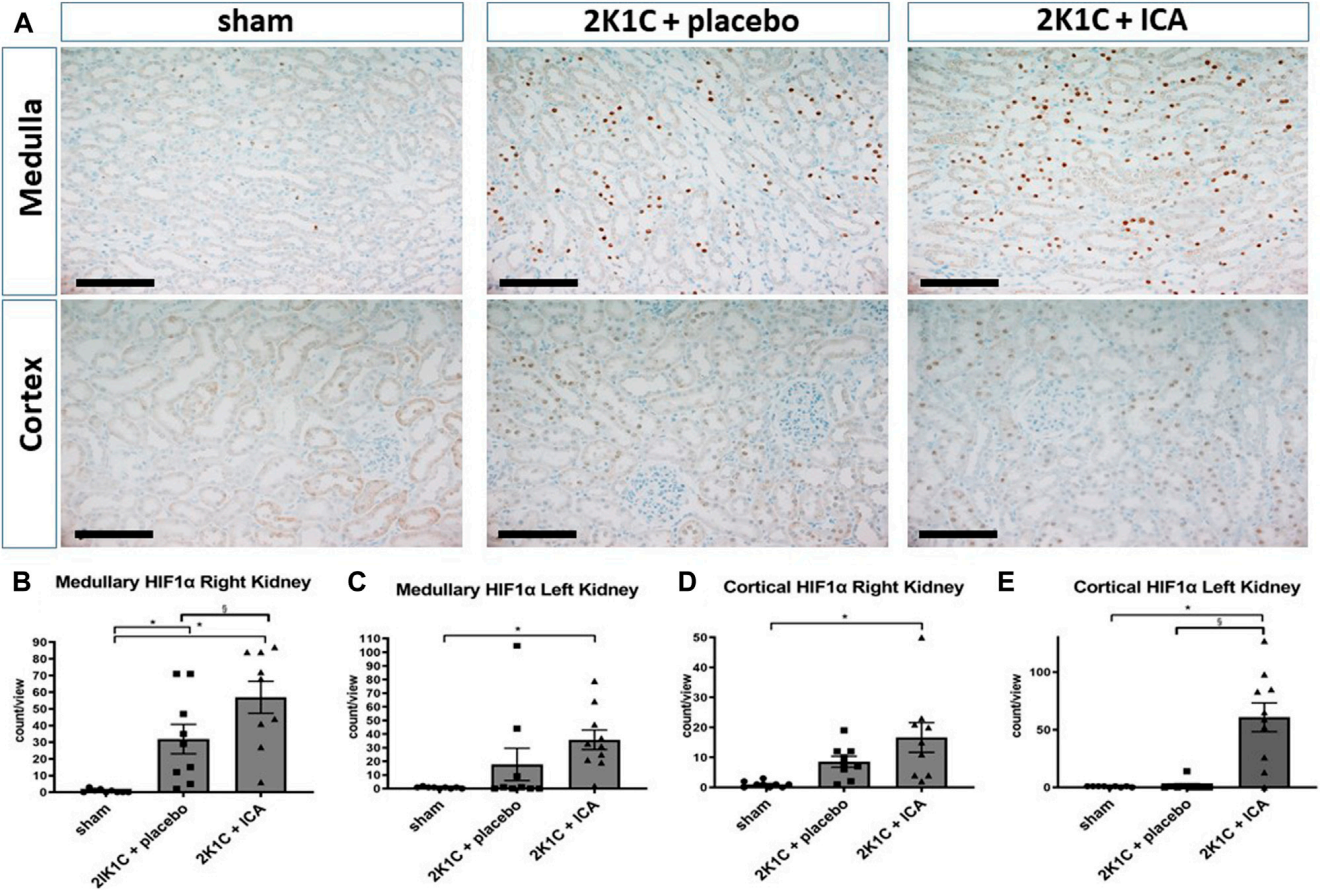
Renal HIF-1α expression 4 h after the last ICA injection at day 19. **(A)**, Exemplary photomicrographs of right kidney tissue stained for HIF-1α. Black scale bar represents 100 µm. Countings of HIF-1α-positive nuclei in the medulla **(B)** and the cortex **(D)** of the right kidney exposed to 2K1C renovascular hypertension as well as in the medulla **(C)** and cortex **(E)** of the left (clipped) kidney. N = 5–8, sham; n = 8–10, 2K1C + placebo; n = 8–10, 2K1C + ICA. 2K1C, 2-kidney-1-clip. ICA, 2-(1-chloro-4- hydroxyisoquinoline-3-carboxamido) acetate. *, *p* < 0.05 versus sham. §, *p* < 0.05 versus 2K1C + placebo. (ANOVA followed by Fisher's Least Significant Differences post-hoc test).

**FIGURE 2:**
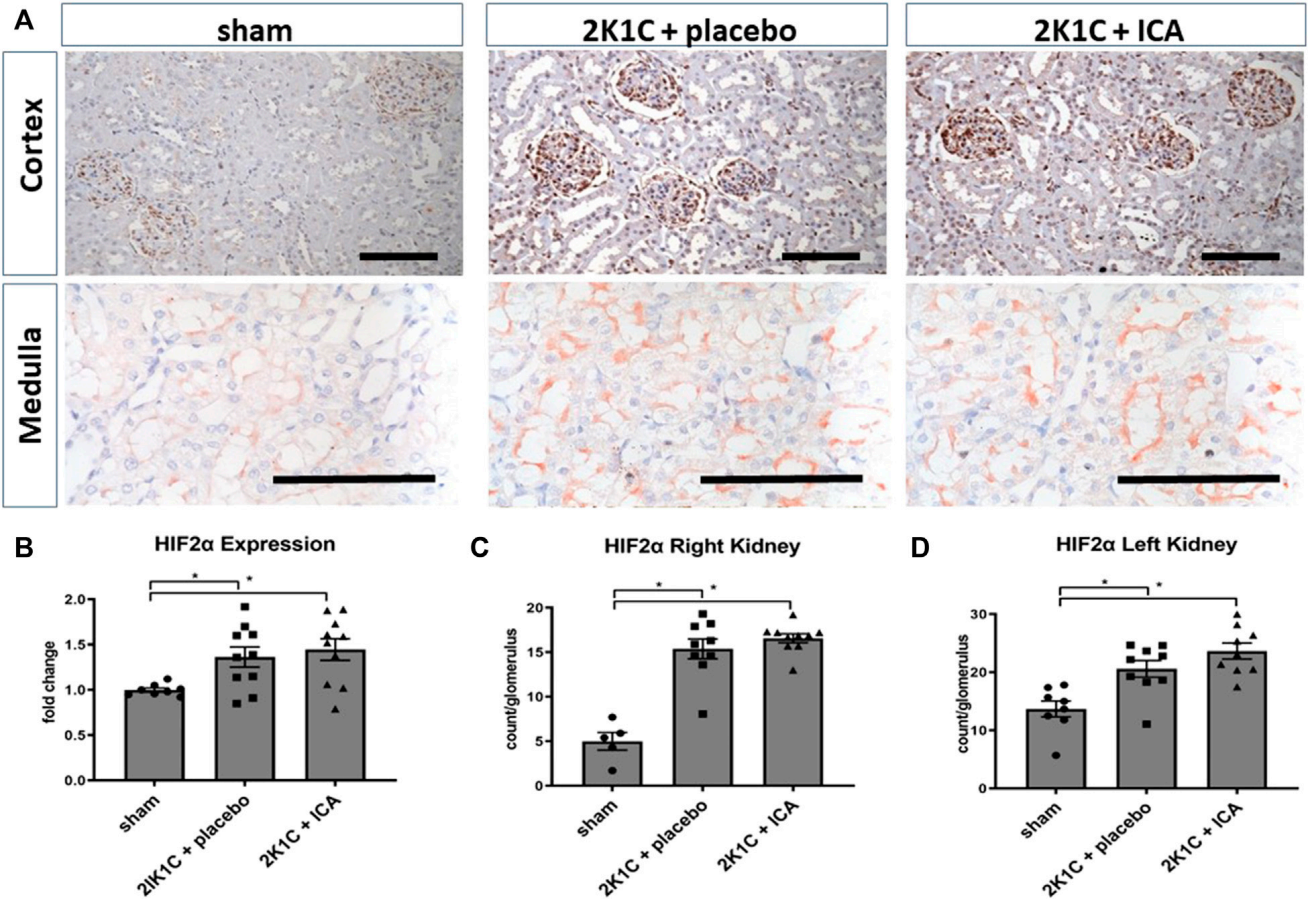
Renal HIF-2α expression 4 h after the last ICA injection at day 19. **(A)**, exemplary photomicrographs of cortical and medullary left kidney tissue stained for HIF-2α; **(B)**, HIF-2 mRNA expression analysis; **(C,D)**, counting of glomerular HIF-2α positive nuclei in the left and right kidney cortex. N = 5–8, sham; n = 8–10, 2K1C + placebo; n = 8–10, 2K1C + ICA. 2K1C, 2-kidney-1-clip renovascular hypertension. ICA, 2-(1-chloro-4- hydroxyisoquinoline-3-carboxamido) acetate. *, *p* < 0.05 versus sham (ANOVA followed by Fisher’s Least Significant Differences post-hoc test), Black scale bar represents 100 µm.

Following the intermittent administration of the HIF PHD inhibitor ICA from day 15 to day 19 after 2K1C operation, HIF-1α stabilization was confirmed at day 19 after 2K1C (4 h after the last ICA injection) in both kidneys. The number of HIF-1α-positive tubular epithelial cells in the medulla (Figure 1B) as well as in the cortex (Figure 1D) of the right kidney (exposed to systemic hypertension) was significantly increased after ICA treatment compared to placebo-treated 2K1C rats. 2K1C-induced hypertension *per se* resulted in modest HIF-1α stabilization in tubular epithelial cells of the right kidney. Similar results were obtained in the medulla and cortex of the left clipped kidneys (Figures 1C,E). Treatment with ICA significantly increased the number of HIF-1α positive tubular cells in the medulla (Figure 1C) and cortex (Figure 1E) of the left clipped kidney, while hypoperfusion *per se* did not significantly stabilize HIF-1α. Tubular HIF-1α stabilization in ICA-treated 2K1C was more prominent in the left than in the right kidney (cortex). Figure 1A; Supplementary Figure S3; Supplementary Figure S4 show exemplary photomicrographs of HIF-1α immunohistochemistry in 2K1C right and left kidneys respectively. In contrast, HIF-2α was significantly stabilized in glomerular cell nuclei of both kidneys after 2K1C, and ICA treatment did not further augment HIF-2α stabilization in our experimental setting (Figure 2). Medullary cell nuclei were only rarely stained in all groups (Figure 2).

**FIGURE 3:**
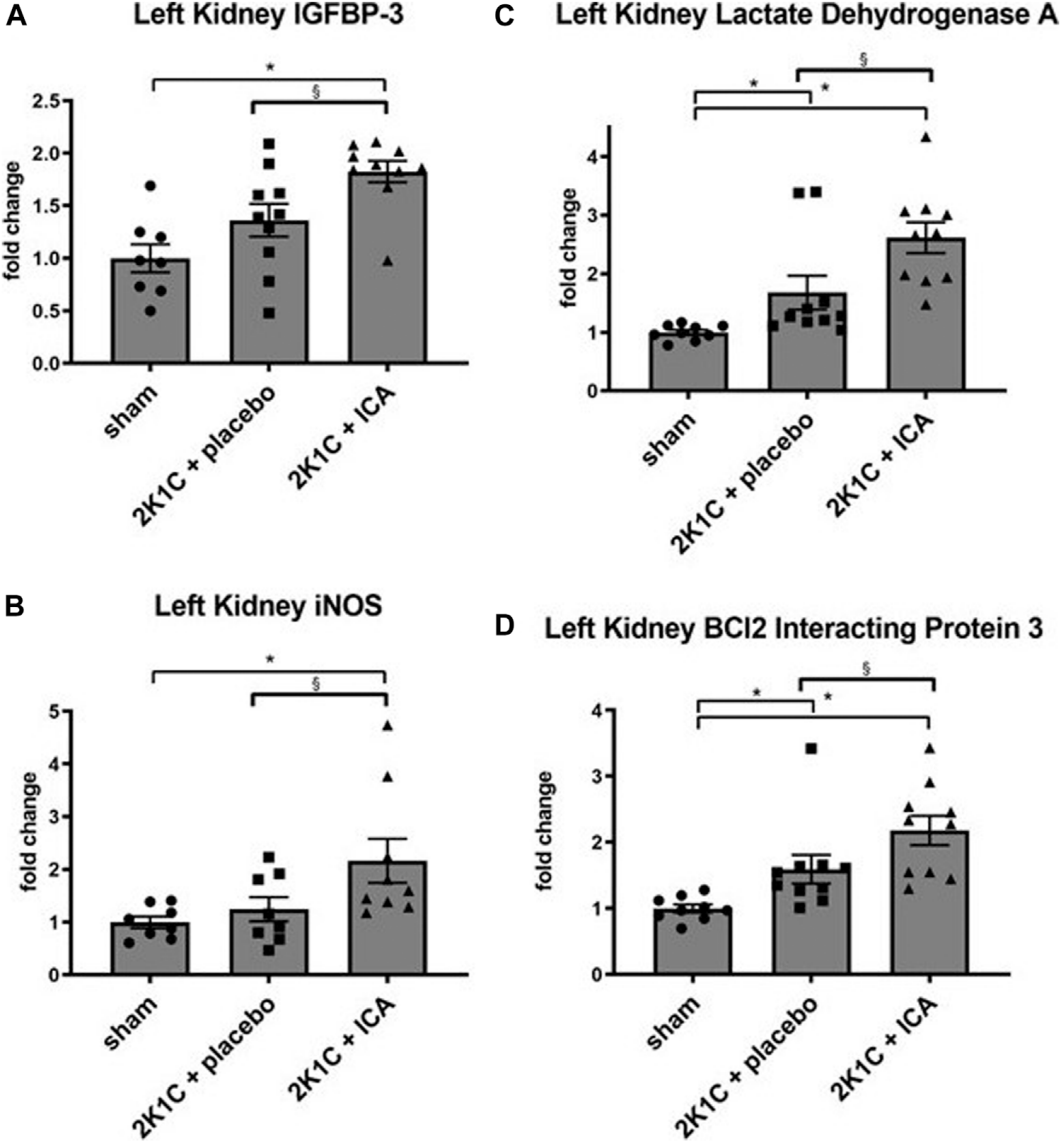
Relative mRNA expression of selected HIF target genes IGFBP3 **(A)**, iNOS **(B)**, Lactat Dehydrogenase A **(C)** and BCl2 Interacting Protein 3 **(D)** induced by ICA treatment in the left (clipped) kidney of 2K1C rats. Measurements were performed 4 h after the last ICA injection at day 19. N = 5–8, sham; n = 8–10, 2K1C + placebo; n = 8–10, 2K1C + ICA. 2K1C, 2-kidney-1-clip renovascular hypertension. ICA, 2-(1-chloro-4- hydroxyisoquinoline-3-carboxamido) acetate. *, *p* < 0.05 versus sham; § *p* < 0.05 versus 2K1C + placebo. (ANOVA followed by Fisher’s Least Significant Differences post-hoc test).

Several HIF target genes were upregulated in both kidneys and the left ventricle of 2K1C animals irrespective of ICA treatment (Supplementary Table S2; Supplementary Table S3; Supplementary Table S4). Moreover, ICA treatment resulted in an additional increase of several HIF target genes 4 hours after administration of the last ICA injection, arguing for augmented HIF activity after ICA treatment. Most target genes were upregulated in the clipped left kidney, while only few were upregulated in the right kidney or the left ventricle exposed to systemic hypertension (Supplementary Table S2; Supplementary Table S3; Supplementary Table S4; Figure 3). Moreover, ICA administration increased the expression of HIF-1-inducible genes (VEGFA, ADM, IGFBP-3, iNOS, BNIP-3, AldoA, LDHA, PFKL, PGK1, PHD2). The expression levels of genes predominantly regulated by HIF-2, like VEGF-R2, Epo, angiopoietin-2, or CCND1, were markedly upregulated by the presence of renovascular hypertension but not further increased by our intermittent treatment regimen (see Supplementary Table S2), although ICA is generally capable of stabilizing HIF-2 (Schley et al., 2012). These findings are in accordance with the notion, that 2K1C renovascular hypertension *per se* predominantly stabilized HIF-2α and the ICA treatment regime predominantly increased HIF-1α stabilization. The intermittent ICA treatment had no systemic effect neither on the course of hematocrit nor on serum erythropoietin levels at day 35 after 2K1C (Table 1).

**TABLE 1:** Course of hematocrit values and erythropoietin levels.

Hematocrit, erythropoietin	Sham	2K1C + placebo	2K1C + ICA
HCT day 15 [%]	41.33 ± 0.73	41.77 ± 0.49	41.95 ± 0.56
HCT day 19 [%]	42.44 ± 0.32	42.56 ± 1.09	43.47 ± 1.55
HCT day 35 [%]	43.71 ± 1.16	44.54 ± 1.39	44.30 ± 1.58
Serum EPO day 35 [ng/ml]	29.69 ± 2.17	70.55 ± 13.23*	80.54 ± 15.46*

*p* < 0.05 versus sham; sham, control sham operation (ANOVA, followed by Fisher’s Least Significant Differences post-hoc test); N = 9, sham; n = 14, 2K1C + placebo; n = 12, 2K1C + ICA., 2K1C, 2-kidney-1-clip hypertensive rats; ICA, 2-(1-chloro-4- hydroxyisoquinoline- 3-carboxamido) acetate; HCT, hematocrit; EPO, erythropoietin.

### ICA treatment did not improve blood pressure and hypertensive organ damage

**FIGURE 4:**
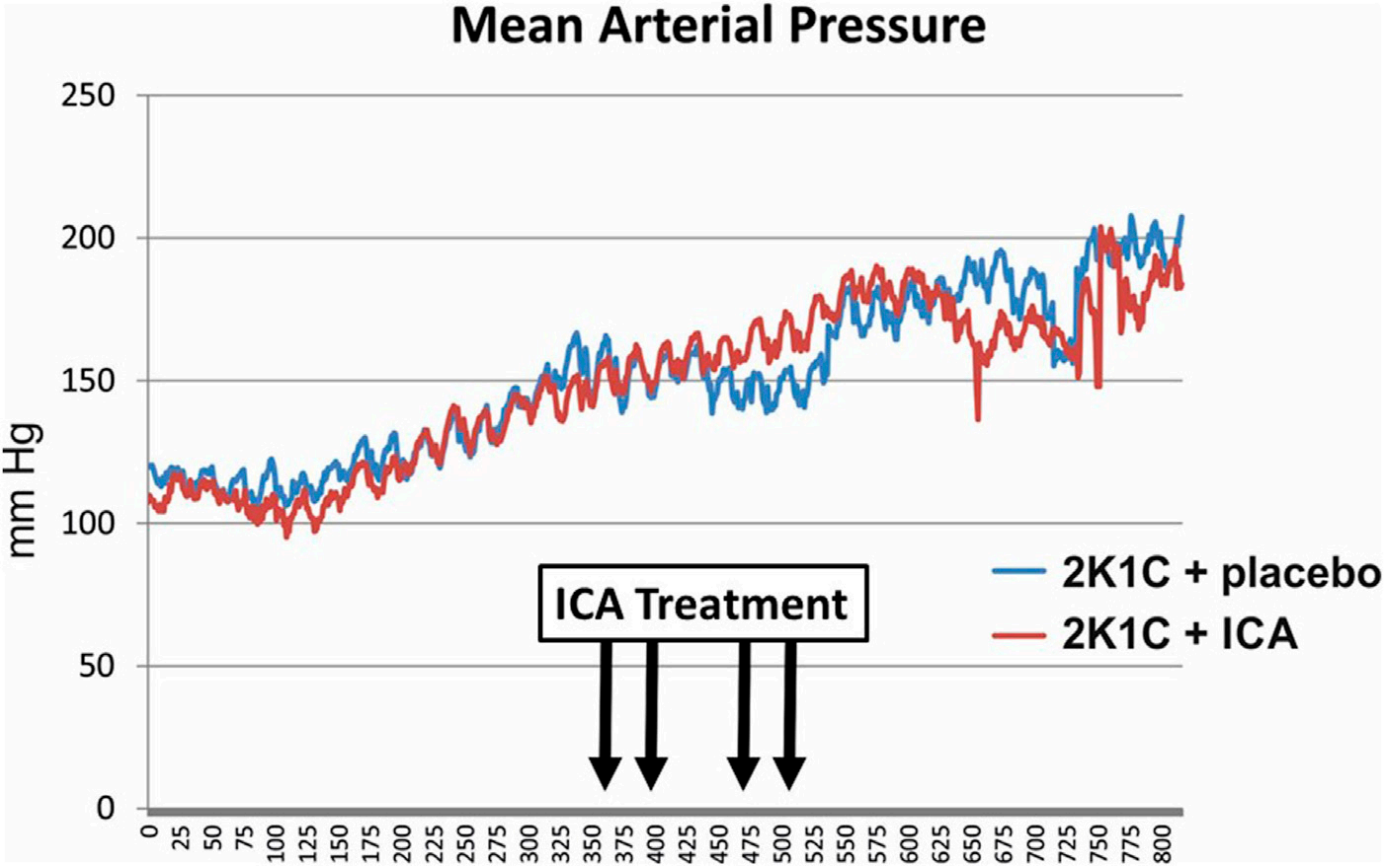
Telemetric recording of mean arterial blood pressure of ICA- and placebo-treated 2K1C hypertensive rats. N = 5 in each group. 2K1C, 2-kidney-1-clip renovascular hypertension. ICA, 2-(1-chloro-4- hydroxyisoquinoline-3-carboxamido) acetate.

As expected, body weights were lower in 2K1C, while relative kidney weights were increased (Table 2). ICA treatment did not influence weight changes (Table 2). Relative left ventricular weights as well as invasively and non-invasively measured blood pressure values were increased 35 days after 2K1C, but again ICA treatment did not modify this increase (Table 2). Telemetric blood pressure measurements revealed a similar course of blood pressure development in placebo- and ICA-treated 2K1C rats (Figure 4). Similar results were obtained by tail-cuff blood pressure measurements (Supplementary Figure S5). Correspondingly, serum aldosterone was augmented by 2K1C, but not significantly altered by ICA treatment (Table 2) While markers of organ damage were not changed by ICA in the right kidney, several markers of organ damage, like KIM-1, IL-11 or iNOS, were further induced by ICA in the clipped left kidney (Table 3). Serum creatinine and urea levels were highest after ICA treatment (Table 2).

**TABLE 2:** Animal weights, blood pressure, serum and urine parameters.

Weights, blood pressure, plasma and urine parameters	Sham	2K1C + placebo	2K1C + ICA
Body weight [g]	388.6 ± 11.2	292.4 ± 11.7*	284.9 ± 10.8*
Rel. right kidney weight [mg/g]	3.15 ± 0.07	5.56 ± 0.33*	5.30 ± 0.25*
Rel. left kidney weight [mg/g]	3.11 ± 0.08	3.82 ± 0.16*	4.02 ± 0.21*
Rel. left ventricular weight [mg/g]	2.20 ± 0.04	3.92 ± 0.15*	3.91 ± 0.11*
MAP intra-arterial [mm Hg]	128.3 ± 9.2	221.1 ± 6.4*	212.3 ± 7.7*
BP tail cuff [mm Hg]	95.6 ± 5.5	208.3 ± 6.7*	196.7 ± 8.9*
Serum aldosterone [ng/ml]	441.0 ± 64.7	3,133.4 ± 561.4*	2943.6 ± 465.6*
Serum creatinine [mg/dl]	0.20 ± 0.03	0.26 ± 0.03	0.48 ± 0.14*
Serum urea [mg/dl]	30.16 ± 3.39	60.05 ± 6.34	100.45 ± 19.55 *^§^
Albuminuria [mg/g creatinine]	0.046 ± 0.002	12.51 ± 2.91*	12.06 ± 3.61*

*, *p* < 0.05 versus sham; §, *p* < 0.05 versus 2K1C + placebo (ANOVA, followed by Fisher’s Least Significant Differences post-hoc test); N = 8–9, sham; n = 15–18, 2K1C + placebo; n = 12–15, 2K1C + ICA., sham, control sham operation; 2K1C, 2-kidney-1-clip hypertensive rats; ICA, 2-(1-chloro-4- hydroxyisoquinoline- 3-carboxamido) acetate; MAP, mean arterial blood pressure; BP, blood pressure.

**TABLE 3:** Markers of organ damage.

	Sham	2K1C + placebo	2K1C + ICA
Right Kidney:			
Glomerulosclerosis [score]	0.66 ± 0.08	1.65 ± 0.09*	1.61 ± 0.14*
Interst. collagen IV [% pos. stain]	4.44 ± 0.52	10.87 ± 1.26*	11.67 ± 1.30*
Interst. Macrophages [no/view]	3.19 ± 0.36	10.33 ± 1.18*	10.06 ± 0.86*
KIM-1 expression [rel. units]	1.00 ± 0.19	237.37 ± 38.13*	411.63 ± 107.57*
Ngal expression [rel. units]	1.00 ± 0.12	22.27 ± 6.15*	20.30 ± 3.89*
Endothelin-1 expr. [rel. units]	1.00 ± 0.07	4.14 ± 0.41*	4.72 ± 0.76*
Adrenomedullin expr. [rel. units]	1.00 ± 0.04	1.29 ± 0.10	1.27 ± 0.13
PAI-1 expression [rel. units]	1.00 ± 0.13	5.49 ± 1.11*	5.73 ± 0.91*
iNOS expression [rel. units]	1.00 ± 0.13	2.99 ± 0.76	4.01 ± 0.82*
eNOS expression [rel. units]	1.00 ± 0.06	1.32 ± 0.07*	1.17 ± 0.10
Left Kidney:			
KIM-1 expression [rel. units]	1.00 ± 0.22	243.27 ± 91.77	1,134.85 ± 521.65*^§^
IL-11 expression [rel. units]	1.00 ± 0.12	2.51 ± 0.30	4.20 ± 0.85*^§^
Endothelin-1 expr. [rel. units]	1.00 ± 0.08	2.10 ± 0.30	3.27 ± 0.63*
iNOS expression [rel. units]	1.00 ± 0.12	1.43 ± 0.34	3.85 ± 1.13*^§^
Left ventricle:			
PAI-1 expression [rel. units]	1.00 ± 0.13	1.69 ± 0.22	2.90 ± 0.24*^§^

*, *p*<0.05 versus sham; §, *p* < 0.05 versus 2K1C + placebo (ANOVA, followed by Fisher’s Least Significant Differences post-hoc test); N = 8-9, sham; n = 15-18, 2K1C + placebo; n = 12-15, 2K1C + ICA., sham, control sham operation; 2K1C, 2-kidney-1-clip hypertensive rats; ICA, 2-(1-chloro-4- hydroxyisoquinoline- 3-carboxamido) acetate.

### ICA treatment did not prevent the development of malignant hypertension

**FIGURE 5:**
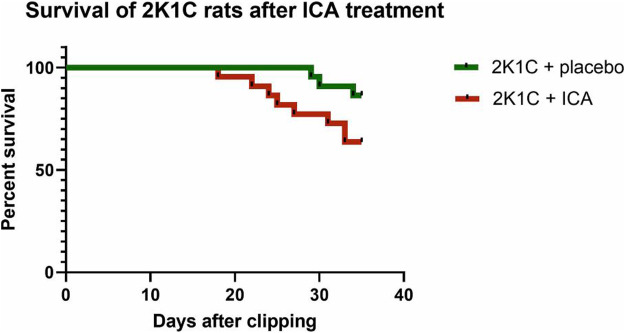
Survival of rats with 2K1C hypertension with or without ICA treatment. 2K1C, 2-kidney-1-clip renovascular hypertension. ICA, 2-(1-chloro-4- hydroxyisoquinoline-3-carboxamido) acetate.

**FIGURE 6:**
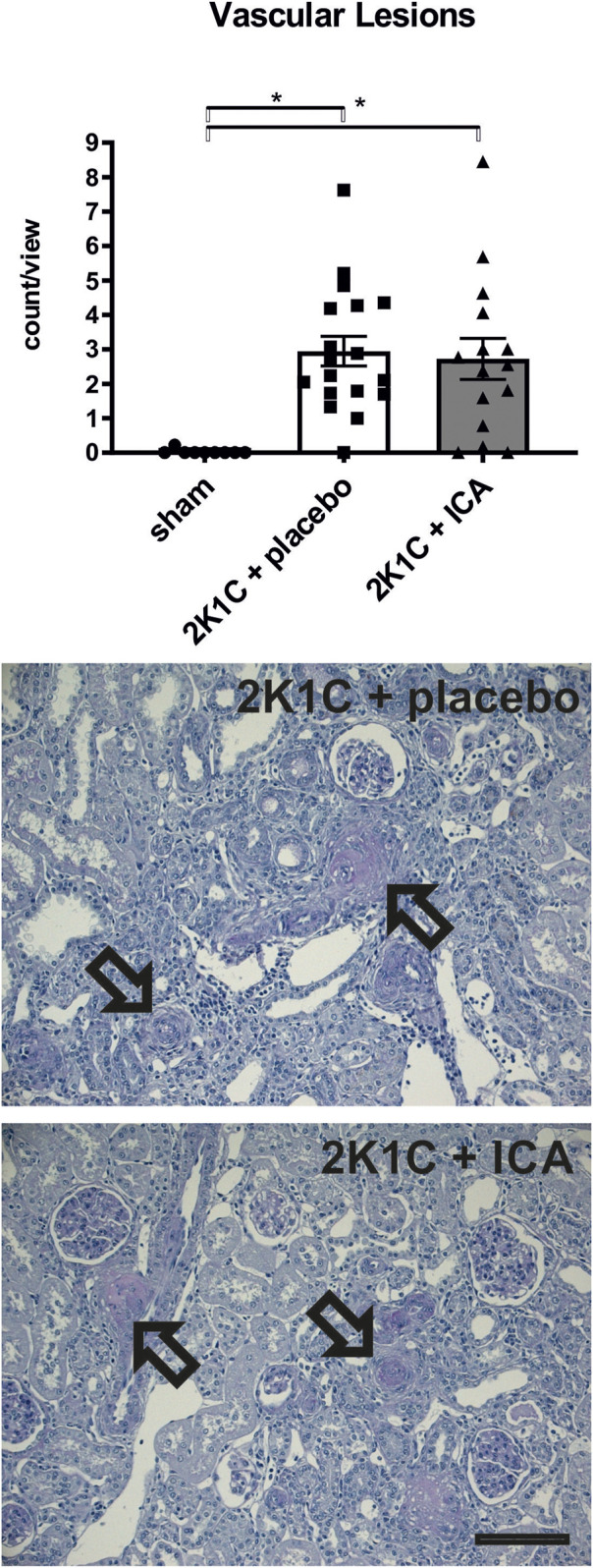
Counting of vascular lesions (onion skin lesions and fibrinoid necroses) in kidney tissue. Representative photomicrographs of PAS-stained sections of kidney tissue exposed to 2-kidney-1-clip (2K1C) renovascular hypertension. Black arrows mark vascular lesions. N = 9, sham; n = 18, 2K1C + placebo; n = 15, 2K1C + ICA. Black bar represents 100 µm. Sham, sham operation. ICA, 2-(1-chloro-4- hydroxyisoquinoline-3-carboxamido) acetate. *, *p* < 0.05 versus sham. (ANOVA followed by Fisher’s Least Significant Differences post-hoc test).

ICA treatment did not prevent mortality during 35 days of the development of hypertension; there was even a trend towards an increased mortality following ICA treatment: From 22 placebo-treated 2K1C rats 3 died, while in the ICA-treated group (n = 22) 8 rats died (Log rank test: *p* = 0.070; Figure 5). Of the surviving 19 placebo-treated 2K1C, 8 developed malignant hypertension according to the criteria mentioned above. Of the 14 surviving ICA-treated 2K1C rats, 6 developed malignant hypertension (*p* = 0.74 χ^2^). 2K1C induced typical vascular lesions (onion skin lesions and fibrinoid necroses), but ICA treatment did not reduce the development of these lesions (Figure 6). As previous studies had shown that renal fibrosis and renal inflammation were augmented in malignant hypertension, the mRNA expression of markers of fibrosis and inflammation was investigated. Collagen I, fibronectin and TGFβ1 expression levels were induced after 2K1C. ICA treatment did not significantly reduce their expression (Supplementary Table S5). The expression levels of the inflammatory markers IL-6, IL-11, MCP-1, CCL-7, CXCL-6, osteopontin, VCAM and ICAM were also induced by 2K1C, but again no reduction was observed after ICA treatment (Supplementary Table S5). Moreover, ICA treatment did not reduce the left ventricular expression of these genes (Supplementary Table S6).

### ICA treatment did not prevent capillary loss

**FIGURE 7:**
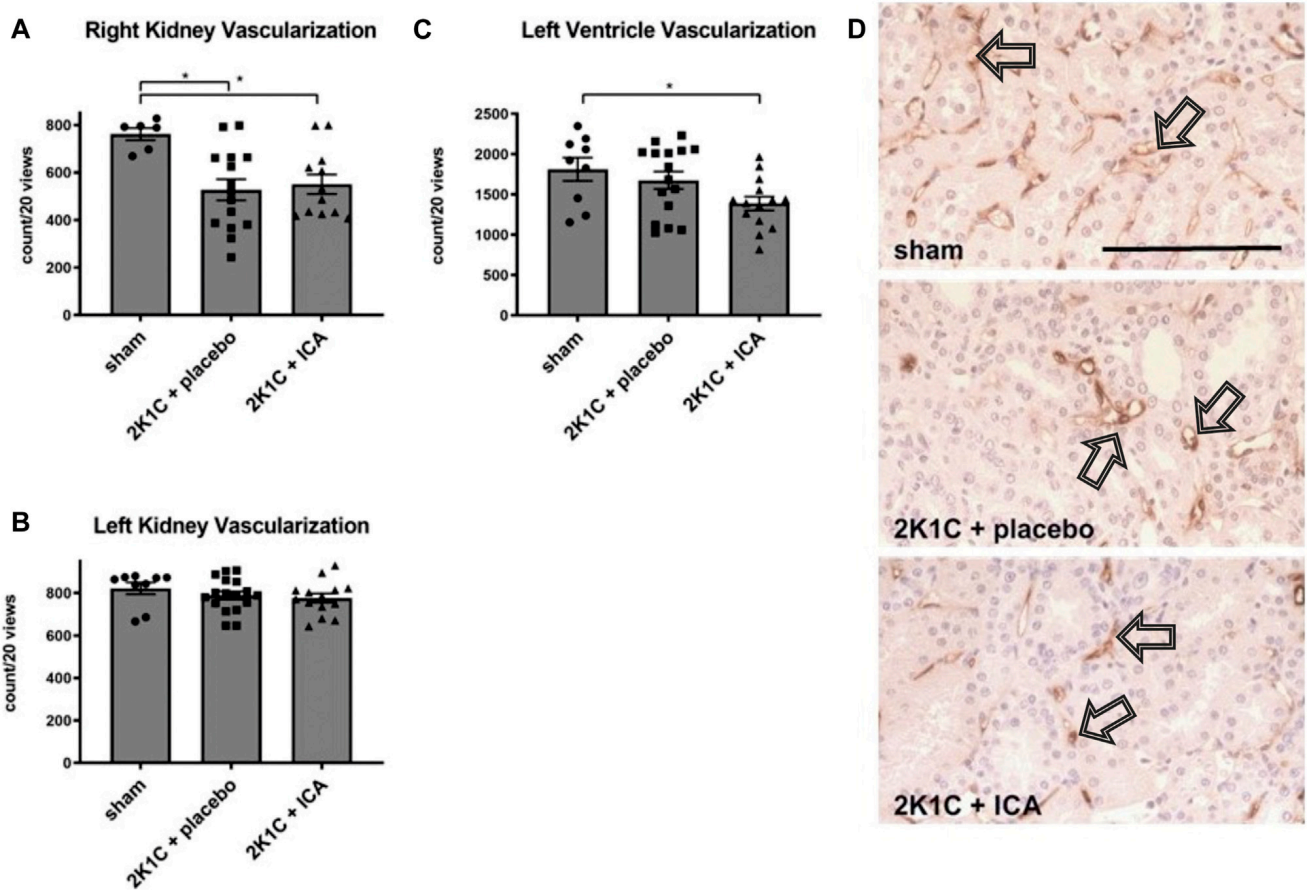
**(A, B, C)**, Counting of vascular cross sections in the left (clipped) and right (exposed to hypertension) kidneys and the left ventricles after staining with aminopeptidase P. N = 8–9, sham; n = 15-16, 2K1C + placebo; n = 12-15, 2K1C + ICA. Sham, sham operation. 2K1C, 2-kidney-1-clip renovascular hypertension. ICA, 2-(1-chloro-4- hydroxyisoquinoline-3-carboxamido) acetate. *, *p* < 0.05 versus sham (ANOVA followed by Fisher’s Least Significant Differences post-hoc test). **(D)** exemplary photomicrographs of endothelial staining (JG12) in right kidney tissue. Black arrows mark stained vascular cross sections. Black scale bar represents 100 µm.

Capillarization of the kidney and the heart was determined at day 35 after 2K1C operation. Some reduction (approximately 20%) of renal capillarization was detected after 2K1C (Figure 7). ICA-treatment did not result in increased capillarization, neither in the kidney, nor in the left ventricle (Figure 8). The expression of angiogenic markers (VEGF-A, VEGF-B, VEGF-C, VEGF-D, angiopoietin-1, angiopoietin-2 and placental growth factor) and respective receptors (VEGF-R1, VEGF-R2, VEGF-R3, Tie-1 and Tie-2) measured at the conclusion of the experiment, 15 days after the termination of ICA treatment, was not increased in renal or left ventricular tissue compared to placebo-treated 2K1C (Supplementary Table S7; Supplementary Table S8).

## Discussion

In the 2K1C rat model of renovascular hypertension, we investigated the effect of HIF stabilization by means of HIF PHD inhibition on the severity of hypertensive target organ damage, especially on the development of malignant hypertension.

HIF-1α expression has been studied in different models of experimental hypertension, and reported results are very variable. Using Western blotting protein expression of HIF-1α was found to be increased under high salt diet (Li et al., 2008; Wang et al., 2010) and in DOCA/salt hypertension (Dallatu et al., 2014) and to be decreased in hypertension induced by nitric oxide synthase inhibition in combination with high salt diet (Dallatu et al., 2013). In Angiotensin II-induced hypertension HIF-1α was detected in glomerular endothelial cells (Luo et al., 2015) and in acute renal neurogenic hypertension in tubular epithelial cells (Koeners et al., 2014). In this study of 2K1C renovascular hypertension HIF-1α stabilization was found in some tubular epithelial cells mainly of the kidney medulla using immunohistochemistry. However, to the best of our knowledge we demonstrate for the first time widespread stabilization of HIF-2α in glomerular cells following 2K1C hypertension, which has not been reported in experimental *systemic* hypertension so far. Interestingly, the development of *pulmonary* hypertension was dependent on HIF-2α in a mouse model (Kapitsinou et al., 2016).

We verified the immunohistochemical detection of HIF-1α and HIF-2α by expression analysis of typical HIF-1 and HIF-2 target genes. HIFα isoforms exhibit cell type- and tissue-specific expression profiles. While HIF-1α is widely expressed across normal tissues and cell types, HIF-2α expression is more restricted (Schodel and Ratcliffe, 2019). In the adult kidney, HIF-2α is strongly expressed in interstitial cells, endothelial cells and glomeruli (Freeburg et al., 2003; Petry et al., 2005; Bernhardt et al., 2006; Paliege et al., 2010; Schietke et al., 2012) whereas in tubular epithelial cells HIF-1α is the predominant isoform (Rosenberger et al., 2002). Furthermore, PHD enzymes also exhibit a distinctive expression pattern in cells and tissues. While PHD-2 is the most widely expressed HIF prolyl hydroxylase, the roles of PHD-1 and PHD-3 in HIF regulation seem to be more tissue-specific (Schodel and Ratcliffe, 2019). To date, the reason and significance for this differential expression profile of HIF-1α and PHD isoforms are still unclear. As HIF-2α has an important role in vascular remodeling and preserves microvascular integrity (Peng et al., 2000; Jiang et al., 2019), the strong expression of HIF-2α in glomerular cells of the unclipped kidney exposed to systemic hypertension might reflect activation of the endothelium and microvascular injury.

In the 2K1C model we previously found (Hartner et al., 2016) that the development of malignant hypertension was associated with impaired angiogenic processes and reduced capillary supply in organs exposed to high blood pressure. Therefore, we aimed to stimulate capillary growth by promoting HIF stabilization using a pharmacological inhibitor of PHDs, as we successfully demonstrated in a rat model of hindlimb ischemia before (Schellinger et al., 2017). When we administered four injections of the PHD inhibitor ICA in the third week after 2K1C and followed the rats for additional 3 weeks, the development of malignant hypertension, however, was not reduced, and typical features of a malignant course (weight loss, renal onion skin lesions and fibrinoid necroses) remained unchanged by the treatment with ICA. Capillary supply of the kidney exposed to high blood pressure was not augmented whereas capillary supply of the left ventricle was even decreased. Finally, signs of fibrosis, inflammation and impaired kidney function, analyzed in an unbiased manner regardless of the category (malignant vs. non-malignant) were also not ameliorated by PHDs inhibition.

Strategies to stabilize HIF as an organ-protective measure have been tested in numerous cardiovascular (Bishop and Ratcliffe, 2015) and kidney (Schodel and Ratcliffe, 2019) diseases; several studies have addressed the effect of HIF stabilization in hypertension. As for HIF expression, data on the effects of PHD inhibitors in hypertension models are inconsistent. Continuously administered dimethyl oxalyl glycine (DMOG) exacerbated hypertension induced by nitric oxide synthase inhibition and high salt diet (Dallatu et al., 2013), but attenuated deoxycorticosterone acetate (DOCA)/salt hypertension (Dallatu et al., 2014) and renal end organ damage in Dahl salt-sensitive rats (Kato et al., 2020), which could be confirmed by silencing of the PHD2 gene in the renal medulla (Zhu et al., 2014). In angiotensin (Ang) II-induced hypertension cobalt chloride did not have an effect on mean arterial pressure and the renal microvasculature but attenuated perivascular fibrosis in coronary arteries (Matsuura et al., 2011). In contrast, daily administration of roxadustat, which was recently approved for the treatment of renal anemia in patients with chronic kidney disease, abolished hypertension and organ injury induced by Ang II (Yu et al., 2021). Genetic deletion of endothelial HIF-1 reduced Ang II-induced hypertension and kidney injury (Luo et al., 2015), whereas deficiency of endothelial HIF-2 accentuated Ang II-induced albuminuria und podocyte lesions without altering the blood pressure (Wang et al., 2014; Luque et al., 2017). (Perim et al., 2018) described that exposure to chronic hypoxia attenuated the development of hypertension in one-kidney, one-clip hypertensive rats.

Thus, the effect of PHD inhibition seems to be highly dependent on the experimental model, clinical context and the specificity of the compounds used. Moreover, the timing of HIF stabilization can also affect the experimental outcome, which might at least partially be explained by temporal differences in the expression of HIFα isoforms (Yu et al., 2012; Kong et al., 2017). So HIF activation clearly mediates beneficial adaptive responses to (acute) hypoxia but under certain, as yet not fully understood conditions it might also support detrimental maladaptive processes in diseases. In this study, markers of tissue injury were significantly enhanced in the clipped kidney of ICA-treated in comparison to placebo-treated 2K1C rats which might imply increased maladaptive response following ICA treatment. However, histological glomerular injury in the right kidney exposed to systemic hypertension and albuminuria were not affected by treatment with ICA.

Other strategies to improve capillary supply in renovascular hypertension, that focused on the poststenotic kidney, were more successful. Saad and coworkers (Saad et al., 2016) demonstrated that adipose-tissue-derived mesenchymal stem cells from patients with renovascular disease grown under hypoxic conditions expressed pro-angiogenic growth factors. In renovascular hypertensive pigs, Chen et al. (Chen et al., 2020) reported an attenuated capillary loss in the poststenotic kidney if stem cells were combined with low-energy shock wave treatment. This latter treatment alone also improved capillary loss and tissue oxygenation in the same porcine model (Zhang et al., 2016). Autologous mesenchymal stem cells infused intraarterially to patients with renovascular disease caused higher renal blood flow in both kidneys (Saad et al., 2017).

In our study, we intended to investigate target organ damage in the absence of potentially confounding effects on blood pressure levels. Neither intraarterial measurements at the end of the experiment in all animals nor long-term radiotelemetric recordings in a smaller sample of animals yielded an effect of HIF PHD inhibition on blood pressure. Vilar and coworkers (Vilar et al., 2008) investigated the effect of chronic hypoxia on blood pressure and several measures of angiogenesis in spontaneously hypertensive rats (SHR). Rats were kept at 10% oxygen for 3–10 weeks which led to stunted growth (30%–40% lower weight in hypoxic animals). Under these conditions, the blood pressure in SHR was identical to that of normotensive control rats, i.e., the development of hypertension was completely abrogated (Vilar et al., 2008). Moreover, muscle perfusion (assessed by microangiography) as well as arteriolar and capillary density were improved by hypoxia. Hematocrit levels are not included in the report by Vilar et al. (2008) but previous studies employing somewhat comparable degrees of hypoxia described hematocrit values as high as 80% (Fisher et al., 1992). Under such conditions, increased capillary flow occurs also in normotensive rats (Fisher et al., 1992).

To avoid large effects on hematocrit values, we applied the HIF PHD inhibitor ICA only intermittently. This therapy did not affect the animals’ body weight, blood pressure or the capillary supply in both kidneys and the left ventricle. We cannot exclude the possibility that daily treatment with the PHD inhibitor would have attenuated hypertension and hypertensive end organ damage. However, the present dose regimen was based on our previous study, which demonstrated an effect on HIF levels and capillaries in skeletal muscle (Schellinger et al., 2017).

One might speculate that capillary growth may respond better to PHD inhibition in case of acute ischemia/hypoxia than in the case of slowly progressive capillary loss and that capillaries may respond better in skeletal muscle than in the heart or kidney. However, we consider an alternative explanation to be more likely. PHD inhibition may not have attenuated malignant hypertension, because renovascular hypertension already resulted in a strong HIF-2α accumulation in glomerular (presumably endothelial) cells, which could not be further increased by the PHD inhibitor.

The necessarily somehow arbitrary definition of malignant versus non-malignant hypertension could be considered as a limitation of our study. Therefore, we did not entirely rely on these categories but instead analyzed all variables as continuous measurements dependent only on the treatment (ICA or placebo) in all renovascular hypertensive rats. Taken together, our results do not support the notion that low-dose, intermittent PHD inhibition alleviates renovascular hypertension, target organ damage or capillary rarefaction in 2K1C rats.

## Data Availability

The original contributions presented in the study are included in the article/Supplementary Material, further inquiries can be directed to the corresponding author.
