# Supplementary material for: No benefit of HIF prolyl hydroxylase inhibition for hypertensive renal damage in renovascular hypertensive rats
Source: Front Physiol. 2023 Jun 26;14:1208105. doi: 10.3389/fphys.2023.1208105 (PMC10331609; doi:10.3389/fphys.2023.1208105)
Supplement: Supplementary file 1 [file DataSheet1.pdf]

List of Supplemental Material

Supplemental Figure 1:

Time line of the experimental procedure. After 2K1C (or sham) operation, weekly measurements of body weights were performed and blood pressure was measured at days 0, 3, 7, 10, 15, 20, 24 and 31 (blue dots). Part of the experimental animals was sacrificed 4 hours after the last ICA treatment for determination of HIF-1 and HIF-2 as well as HIF target genes. The other rats were sacrificed 2 weeks after the last ICA treatment for evaluation of angiogenesis, inflammation, fibrosis and renal outcome. The yellow and red fields symbolize the development of hypertension (yellow) and the occurrence of malignant lesions (red) as we expected it to occur when we designed the experiment. We estimated that before day 14 all 2K1C rats were hypertensive, and that after day 14 malignant lesions would start to occur in some of the 2K1C rats.

Supplemental Figure 2:

Exemplary photomicrographs of glomeruli displaying score 0 to 4 for the semiquantitative evaluation of glomerulosclerosis. Black bar represents 50 µm

Supplemental Figure 3:

Exemplary photomicrographs of left renal tissue stained for HIF-1α 4 hours after the last ICA injection at day 19. Black bar represents 100 µm

Supplemental figure 4:

Exemplary photomicrographs of right renal tissue from ICA treated 2K1C rats 4 hours after termination of ICA treatment in higher magnification show localization of HIF-1α and HIF-2α. Black bar represents 50 µm

Supplemental figure 5:

Tail-cuff blood pressure measurements at days 0, 3, 7, 10, 15, 20, 24 and 31 after 2K1C operation.

Supplemental Table 1:

List of primer pairs used in the study

Supplemental Table 2:

HIF target genes in the left kidney 4 hours after administration of the last ICA dose. N=8, sham; n=9-10, 2K1C + placebo; n=9-10, 2K1C + ICA. *, p<0.05 versus sham; § p<0.05 versus 2K1C + placebo (ANOVA followed by Fisher's Least Significant Difference post-hoc test)

Supplemental Table 3:

HIF target genes in the right kidney 4 hours after administration of the last ICA dose. N=8, sham; n=9-10, 2K1C + placebo; n=9-10, 2K1C + ICA. *, p<0.05 versus sham; § p<0.05 versus 2K1C + placebo (ANOVA followed by Fisher's Least Significant Difference post-hoc test)

Supplemental Table 4:

HIF target genes in the left ventricle 4 hours after administration of the last ICA dose. N=8, sham; n=9-10, 2K1C + placebo; n=9-10, 2K1C + ICA. *, p<0.05 versus sham; § p<0.05 versus 2K1C + placebo (ANOVA followed by Fisher's Least Significant Difference post-hoc test)

Supplemental Table 5:

Right renal expression of markers of tissue fibrosis and inflammation. N=8-9, sham; n=15-18, 2K1C + placebo; n=12-15, 2K1C + ICA. *, p<0.05 versus sham (ANOVA followed by Fisher's Least Significant Difference post-hoc test). There were no significant differences between 2K1C-ICA and 2K1C-placebo.

Supplemental Table 6:

Left ventricular expression of markers of tissue fibrosis and inflammation. N=8-9, sham; n=15-18, 2K1C + placebo; n=12-15, 2K1C + ICA. *, p<0.05 versus sham (ANOVA followed by Fisher's Least Significant Difference post-hoc test) There were no significant differences between 2K1C-ICA and 2K1C-placebo.

Supplemental Table 7:

Right renal expression of angiogenetic markers. N=8-9, sham; n=15-18, 2K1C + placebo; n=12-15, 2K1C + ICA. *, p<0.05 versus sham; §, p<0.05 versus 2K1C + placebo. (ANOVA followed by Fisher's Least Significant Difference post-hoc test)

Supplemental Table 8:

Left ventricular expression of angiogenetic markers. N=8-9, sham; n=15-18, 2K1C + placebo; n=12-15, 2K1C + ICA. *, p<0.05 versus sham; §, p<0.05 versus 2K1C + placebo. (ANOVA followed by Fisher's Least Significant Difference post-hoc test)

Supplemental Figure 1

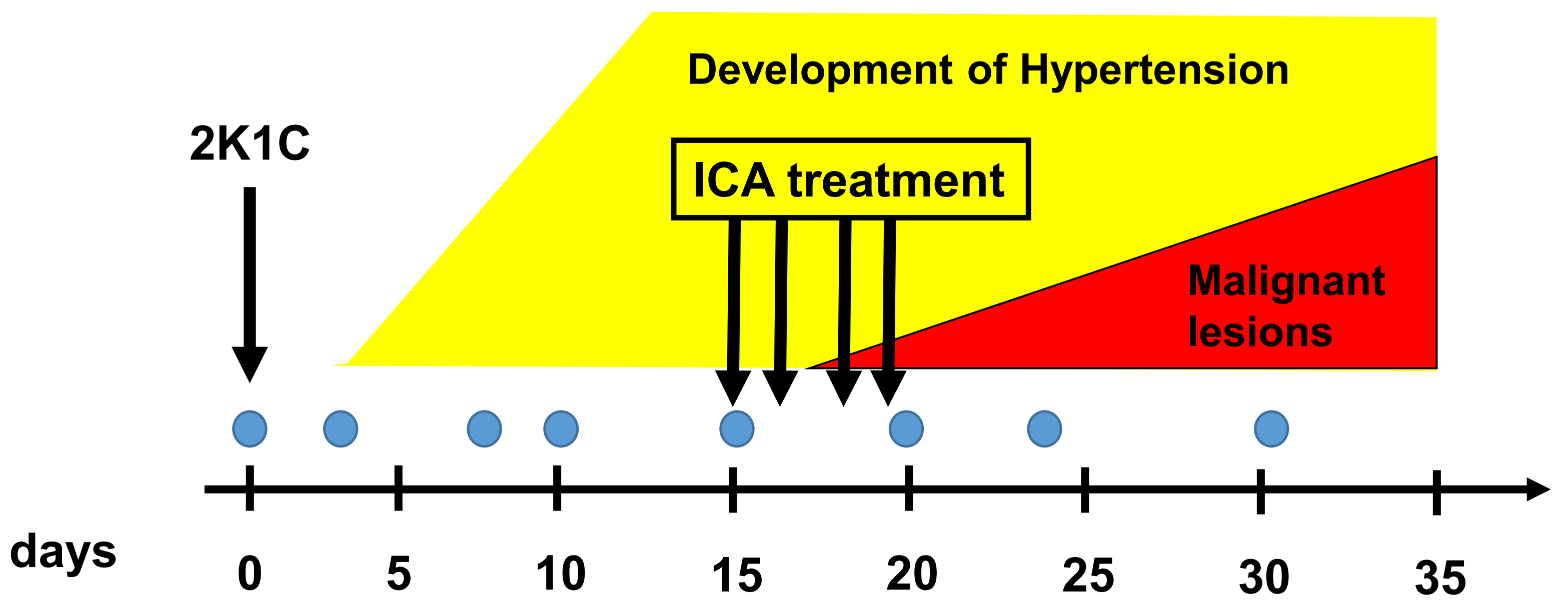

Supplemental Figure 2

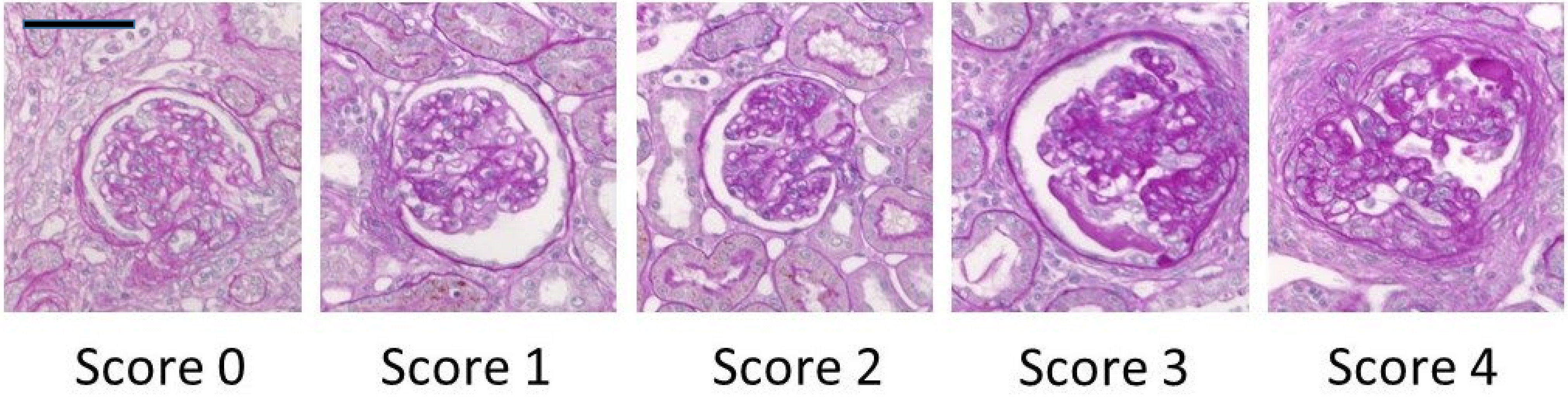

Supplemental Figure 3

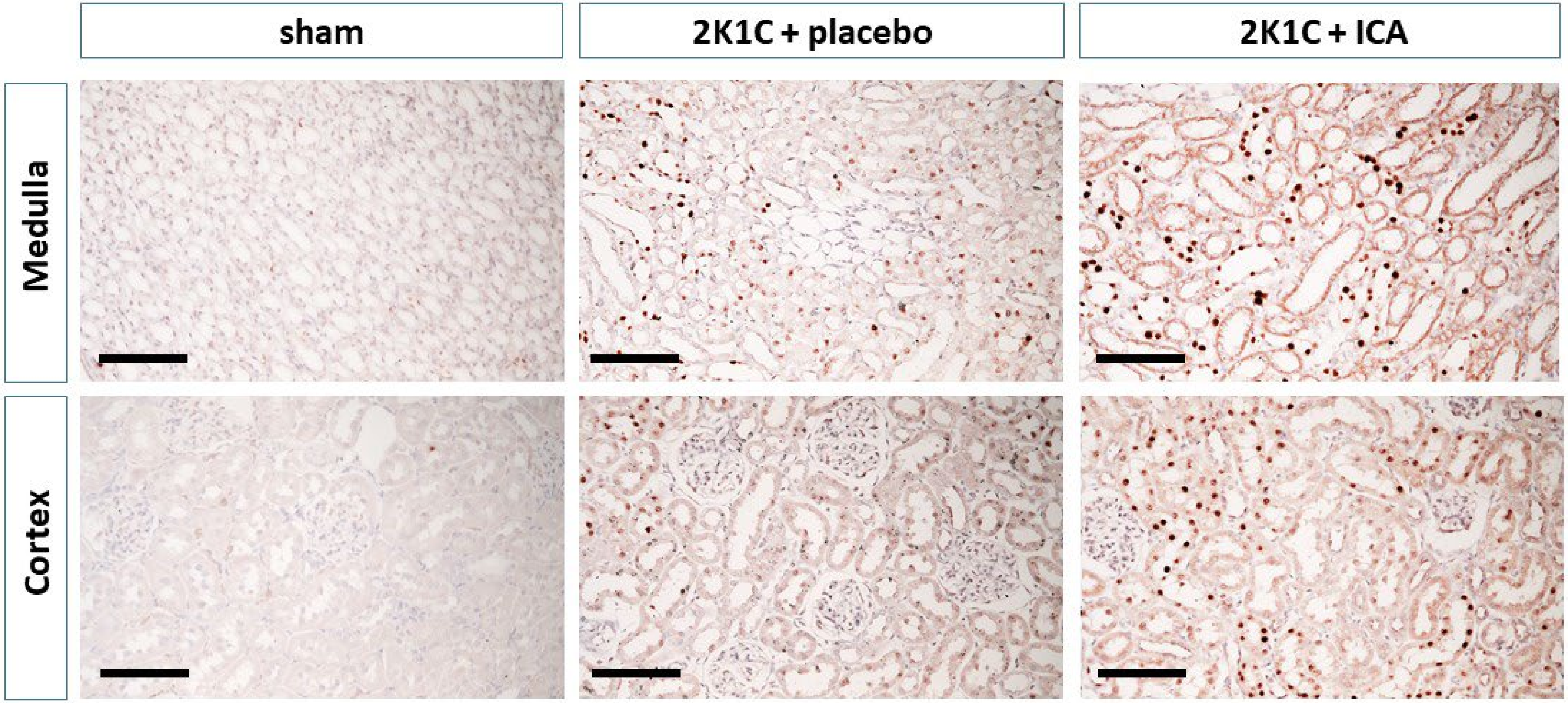

Supplemental Figure 4

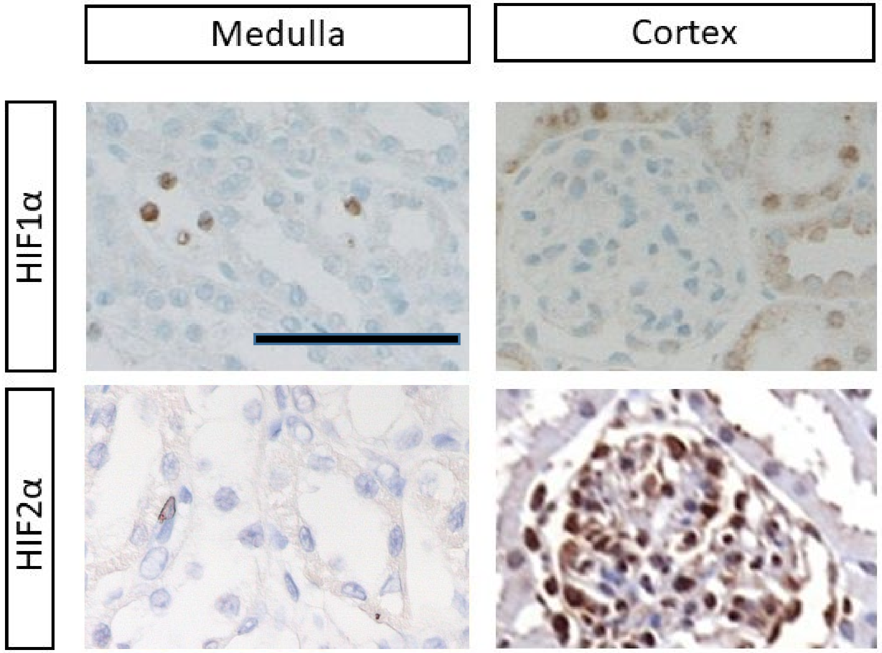

Supplemental Figure 5

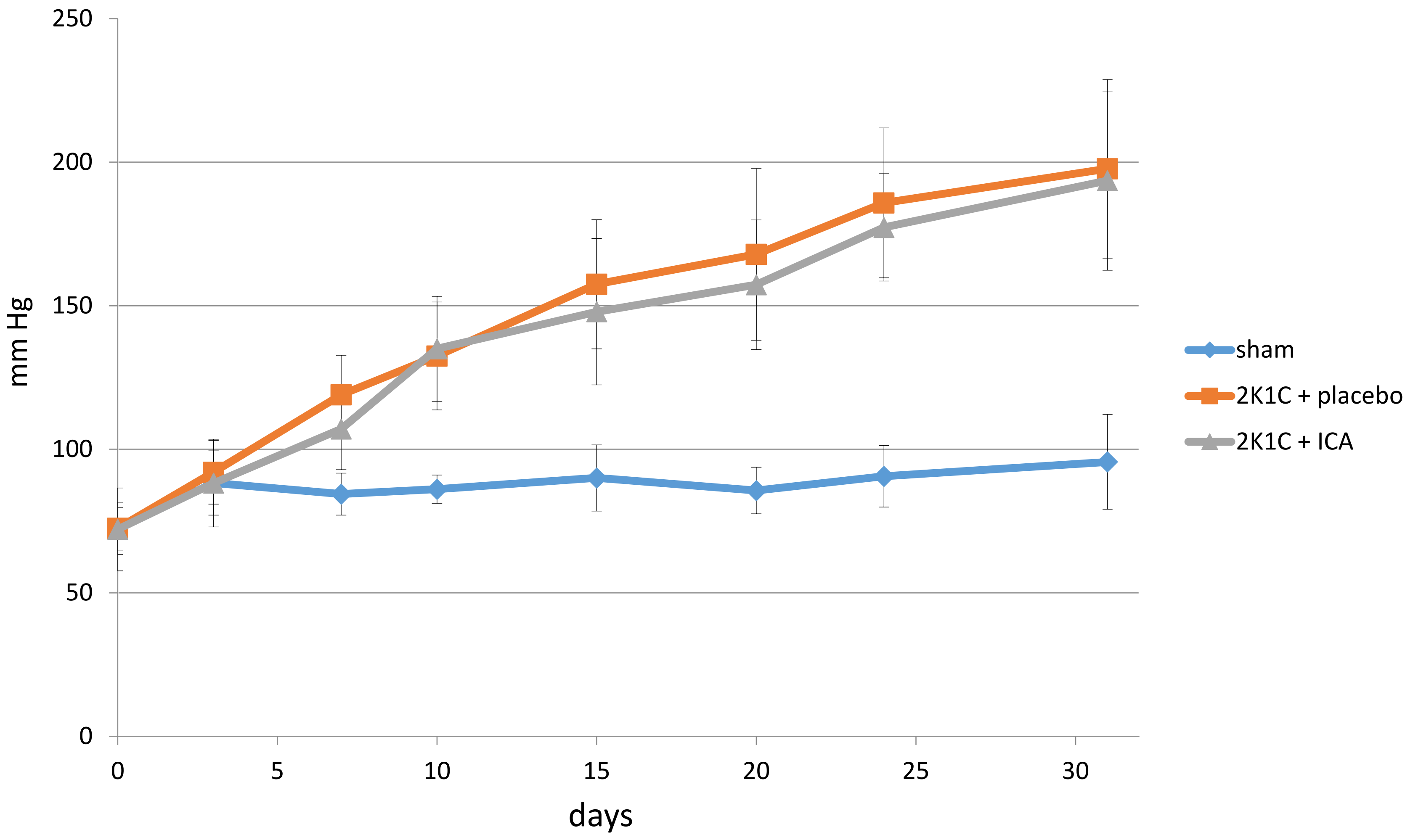

Supplemental Table 1: List of primer pairs used in the study

	Forward	Reverse
ADM	5'- TCA GCA GGG TAT CGG AGC AT -3'	5'- GCG AGC GAA CCC AAT AAC A -3'
AldoA	5'- CAC TGT ACC AGA AGG CAG ATG ATG -3'	5'- CTT ATC TAC CTT AAT GCC CAC AAC AC -3'
AldoC	5'- CAC TCT ACC AGA AAG ATG ACA ATG GT -3'	5'- TTG TCA ACC TTG ATG CCT ACG A -3'
AP-1	5'- AAA CCA CAC GGC CAC CAT -3'	5'- TGG ATT TCA AGA CGG GAT GT -3'
	probe: TGG AGA TAG GAA CCA GCC TCT TGT CTC AGA CT	
AP-2	5'- GAC CAG TGG GCA TCG CTA CG -3'	5'- CAT TGT CCG AAT CCT TTG TGC T -3'
	probe: AAG GCA GCG AGG CAC ACT CTC TGT ATG AG	
BNIP-3	5'- TAG CCA TTG GAT TGG GGA TCT -3'	5'- AGT GGA AGT TGT CAG ACG CC -3'
CA-9	5'- AGA AAT CGC AGA GGA AGG CTC -3'	5'- TAG TAG CGA CTG AGG TCC GA -3'
CCL-5	5'- GTC GTC TTT GTC ACT CGA AGG A -3'	5'- GAT GTA TTC TTG AAC CCA CTT CTT CTC -3'
	probe: CCG CCA AGT GTG TGC CAA CCC	
CCL-7	5'- GCC GCG CTT CTG TGT GT -3'	5'- TGG ATG AAT TGG TCC CAT CTG -3'
	probe: CTG CTC ACA GCT GCT GCT TTC ACC G	
CCND1	5'- GGG CAG CCC CAA CAA CTT -3'	5'- GCA GTC CGG GTC ACA CTT G -3'
CXCL-6	5'- CAC ACT GCC ACA GCA TCG A -3'	5'- CAG CGT AGC TCC GTT GCA A -3'
Collagen I	5'-AGA GCG GAG AGT ACT GGA TCG A-3'	5'-CTG ACC TGT CTC CAT GTT GCA-3'
Endothelin-1	5'- CCT TTG CAG AAT GGA TTA TTT TCC -3'	5'- TGC TCC CAA GAC AGC TGT TTC -3'
eNOS	5'- GGA CCC AAG TTT CCT CGA GTA A -3'	5'- GGA TCC CAA GCA GCG TCT T -3'
	probe: CAG CAT CAC CTA CGA TAC CCT CAG TGC A	
EPAS	5'- TTG GCC GCT CTG CCT ATG -3'	5'- CCC CTT GGT GCA CAA GTT CT -3'
EPO	5'- CCG TCC CAG ATA CCA AAG TCA -3'	5'- TTG CCA AAC TTC TAC AGC CTG TT -3'
Fibronectin	5'-TTG CAA CCC ACC GTG GAG TAT GTG-3'	5'-CTC GGT AGC CAG TGA GCT TAA CAC-3'
HIG-2	5'-GCA TCA GCC ATG CTT TGA CA-3'	5'-CTG CCA GGC CTA GTA ACA GT-3'
HO-1	5'-GCC TGC TAG CCT GGT TCA AGA-3'	5'-GAG TGT GAG GAC CCA TCG CA-3'
ICAM-1	5'- GGG CCC CCT ACC TTA GGA A -3'	5'- GGG ACA GTG TCC CAG CTT TC -3'
IGFBP-3	5'-GTG CGC TTA GTT CTC CGG ATG AC-3'	5'-CCA GGG TCT CCA ACA AGG AAG ATG-3'
IL-1b	5'- GACCTGTTCTTTGAGGCTGACA -3'	5'- CTCATCTGGACAGCCCAAGTC -3'
IL-6	5'-GCC CTT CAG GAA CAG CTA TGA-3'	5'-TGT CAA CAA CAT CAG TCC CAA GA-3'
	probe: TCT CCG CAA GAG ACT TCC AGC CAG TT	
IL-11	5'- ATC TGG ATA GCG CTG TCC TCT T -3'	5'- TTG TCT CTC ATC TGT GCA GCT AGT T -3'
	probe: CAG GTC CCT CCT GGC AGA CAC ACG	
iNOS	5'- CCT TCA GGT ATG CGG TAT TTG G -3'	5'- AGG CTC CCA GGT GAG ACA GTT -3'

	probe: TGC CTT TGC TCA TGA CAT CGA CCA G	
KIM-1	5′-ATA ATC ACA CTG TAA GAA TCC CTT TGA G-3′	5′-CAA CGG ACA TGC CAA CAT AGA-3′
LDHA	5′- CAA ACC CAG TGG ATA TCT TGA CCT A -3′	5′- CGA ATC CAG ATT GCA ACC ACT T -3′
MCP-1	5′-CCT CCA CCA CTA TGC AGG TCT C-3′	5′-GCA CGT GGA TGC TAC AGG C-3′
	probe: TCA CGC TTC TGG GCC TGT TGT TCA	
Ngal	5′-TCA CCC TGT ACG GAA GAA CCA-3′	5′-ACT TGG CAA AGC TGA CGA ATC-3′
OPN	5′-AAA GTG GCT GAG TTT GGC AG-3′	5′-AAG TGG CTA CAG CAT CTG AGT GT-3′
	probe: TCA GAG GAG AAG GCG CAT TAC AGC A	
PAI-1	5′- GTT CAC CAC TCC GGA TGG G -3′	5′- TGG TAG GGC AGT TCC AGG AT -3′
PFKL	5′- TGA TTG GCT GTT CAT CCC TG -3′	5′- GAG ATA GGC TTT CCA TGC CG -3′
PGK1	5′- CCT ATG AAG AAC AAC CAG ATA ACG AA -3′	5′- CTC CAT TGT CCA AGC AGA ATT TG -3′
PHD2	5′- ATA CGC CAC AAG GTA CGC AA -3′	5′- TCG CTC GTC TGC ATC GAA AT -3′
PlGF	5′-CCT GTC TGC TGG GAA CAA CT-3′	5′-CAT TGA AAG GCA CCA CTT CCA-3′
PKM	5′- TGA AGT ACG CCC GAG GAT CT -3′	5′- GAA TGA AGG CAG TCC CTG CT -3′
TGF-β1	5′-TGG AAG TGG ATC CAC GCG CCC AAG G-3′	5′-GCA GGA GCG CAC GAT CAT GTT GGA C-3′
Tie1	5′- GCC CCA GGA CAG CAT GAT TA-3′	5′- TCT CAC TGG GAT CCA CCA CA -3′
Tie2	5′-GCT GGA AGA ACG AAA GAC ATA CG -3′	5′- GCT CTC GTG CCA GTG AAG AGA -3′
TNF-α	5′-ATG GGC TCC CTC TCA TCA GT-3′	5′-GCT TGG TGG TTT GCT ACG AC-3′
VCAM	5′- TGT GGA AGT GTG CCC GAA AT -3′	5′- TGC CTT GCG GAT GGT GTA C -3′
VEGF-A	5′- AAC GAA AGC GCA AGA AAT CC -3′	5′- GCT CAC AGT GAA CGC TCC AG -3′
VEGF-B	5′- TGT ACC CAG GCC CCT GTG T -3′	5′- GCA CGT GCA TAA ACA TCT ATC CA -3′
VEGF-C	5′- CAG CAA GAC GTT GTT TGA AAT TAC A -3′	5′- GTG ATT GGC AAA ACT GAT TGT GA -3′
VEGF-D	5′- ACA CCG AGC AGT GAA GGA TG -3′	5′- AAC ACA GAC CGG GAT GAT CG -3′
VEGF-R1	5′- CGA CAC TCT TTT GGC TCC TTC TAA C -3′	5′- TGA CAG GTA GTC CGT CTT TAC TTC G -3′
VEGF-R2	5′- CCA CCC CAG AAA TGT ACC AAA C -3′	5′- AAA ACG CGG GTC TCT GGT T -3′
VEGF-R3	5′- CAT TGT GCA CGA AAA GCC CT -3′	5′- CGG GTA GCT TCA CCA TCT CG -3′
18S	5′- TTG ATT AAG TCC CTG CCC TTT GT -3′	5′- CGA TCC GAG GGC CTC ACT A -3′

ADM, adrenomedullin; AldoA/AldoC, Aldolase A/C; AP, angiopoietin; BNIP-3, BCL2 interacting protein 3; CA-9, Carbonic anhydrase 9, CCL-5, Rantes; CCL-7, MCP-3; CCND1, Cyclin D1; eNOS, endothelial NO synthase; EPAS, HIF-2a; EPO, erythropoietin; HIG-2, hypoxia induced gene-2=HILPDA; HO-1, hemeoxigenase-1; ICAM, intercellular adhesion molecule; IGFBP, insulin-like growth factor binding protein; IL, interleukin; iNOS, inducible NO synthase; KIM-1, kidney injury molecule-1; LDHA, Lactate dehydrogenase A; MCP-1, macrophage chemoattractant protein-1= CCL2; Ngal, neutrophil gelatinase-associated lipocalin; OPN, osteopontin, PAI-1, plasminogen activator inhibitor-1; PFKL, Phosphofructokinase liver type; PGK1, Phosphoglycerate kinase 1; PHD2, Prolyl hydroxylase domain containing protein 2, PlGF, placental growth factor; PKM, Pyruvate kinase M1/M2; TGF, transforming growth factor; TNF, tumor necrosis factor; VCAM, vascular cell adhesion molecule; VEGF, vascular endothelial growth factor, VEGF-R, vascular endothelial growth factor receptor.

Supplemental Table 2: HIF Targets in left kidney 4 hours after administration of ICA

mRNA expression	sham	2K1C + placebo	2K1C + ICA
EPO [rel. units]	1.00±0.15	9.74±6.11	10.73±4.14
HO-1 [rel. units]	1.00±0.55	1.27±0.63	4.12±2.49
HIG-2 [rel. units]	1.00±0.05	1.72±0.36	1.89±0.33
VEGFA [rel. units]	1.00±0.03	1.69±0.06 *	2.00±0.07 *§
VEGF-R1 [rel. units]	1.00±0.07	1.65±0.11 *	1.74±0.07 *
VEGF-R2 [rel. units]	1.00±0.07	1.44±0.10 *	1.60±0.08 *
AP-1 [rel. units]	1.00±0.13	1.24±0.12	1.36±0.16
AP-2 [rel. units]	1.00±0.08	1.46±0.10 *	1.57±0.09 *
PlGF [rel. units]	1.00±0.06	1.94±0.19*	1.50±0.19 *
IGFBP-3 [rel. units]	1.00±0.13	1.36±0.15	1.83±0.10 *§
ADM [rel. units]	1.00±0.05	1.43±0.10 *	1.88±0.12 *§
ET-1 [rel. units]	1.00±0.07	3.27±1.73	1.96±0.40
PAI-1 [rel. units]	1.00±0.13	1.84±0.69	1.21±0.23
iNOS [rel. units]	1.00±0.11	1.25±0.23	2.16±0.42 *§
AldoA [rel. units]	1.00±0.06	1.58±0.10 *	1.89±0.09 *§
AldoC [rel. units]	1.00±0.08	1.82±0.25 *	1.84±0.10 *
BNIP-3 [rel. units]	1.00±0.06	1.59±0.21 *	2.18±0.22 *§
CA-9 [rel. units]	1.00±0.24	1.12±0.22	1.23±0.32
CCND1 [rel. units]	1.00±0.11	1.15±0.13	1.20±0.13
LDHA [rel. units]	1.00±0.04	1.68±0.28	2.62±0.26 *§
PFKL [rel. units]	1.00±0.05	1.86±0.23 *	2.60±0.20 *§
PGK1 [rel. units]	1.00±0.09	1.79±0.13 *	2.07±0.06 *§
PHD2 [rel. units]	1.00±0.06	1.30±0.06 *	1.47±0.05 *§
PKM [rel. units]	1.00±0.04	1.09±0.06	1.12±0.02

*, $p<0.05$ versus sham; § $p<0.05$ versus 2K1C; sham, control sham operation; 2K1C, 2-kidney-1-clip hypertensive rats; ICA, 2-(1-chloro-4- hydroxyisoquinoline- 3-carboxamido) acetate; EPO, erythropoietin; HO, heme oxigenase; HIG, HIF-inducible gene; VEGF, vascular endothelial growth factor; VEGF-R, vascular endothelial growth factor receptor; AP, angiopoietin; PlGF, placental growth factor; IGFBP, insulin like growth factor binding protein; ADM, adrenomedullin; ET, endothelin; PAI, platelet activator inhibitor; iNOS, inducible NO synthase; Aldo, aldolase; BNIP-3, BCL2 interacting protein 3; CA-9, Carbonic anhydrase 9; CCND1, Cyclin D1; LDHA, Lactate dehydrogenase A; PFKL, Phosphofructokinase liver type; PGK1, Phosphoglycerate kinase 1; PHD2, Prolyl hydroxylase domain containing protein 2; PKM, Pyruvate kinase M1/M2.

Supplemental Table 3: HIF Targets in right kidney 4 hours after administration of ICA

**mRNA expression**	**sham**	**2K1C + placebo**	**2K1C + ICA**
EPO [rel. units]	1.00±0.12	5.91±2.89	10.94±3.91 *
HO-1 [rel. units]	1.00±0.51	2.31±1.58	3.00±2.13
HIG-2 [rel. units]	1.00±0.07	1.24±0.10	1.49±0.11 *
VEGFA [rel. units]	1.00±0.05	1.75±0.10 *	1.95±0.20 *
VEGF-R1 [rel. units]	1.00±0.08	1.14±0.13	1.44±0.23
VEGF-R2 [rel. units]	1.00±0.06	1.13±0.09	1.43±0.24
AP-1 [rel. units]	1.00±0.13	1.08±0.17	1.29±0.18
AP-2 [rel. units]	1.00±0.12	1.76±0.19 *	1.99±0.28 *
IGFBP-3 [rel. units]	1.00±0.05	0.90±0.08	1.19±0.13 §
ET-1 [rel. units]	1.00±0.08	1.34±0.21	1.77±0.29 *
ADM [rel. units]	1.00±0.04	1.49±0.12 *	1.68±0.11 *
iNOS [rel. units]	1.00±0.10	1.09±0.20	1.87±0.29 *§
AldoA [rel. units]	1.00±0.05	1.42±0.13 *	1.71±0.11 *
AldoC [rel. units]	1.00±0.07	1.27±0.25	1.62±0.23 *
BNIP-3 [rel. units]	1.00±0.07	1.39±0.23	2.00±0.26 *§
CA-9 [rel. units]	1.00±0.15	1.29±0.22	1.68±0.48
CCND1 [rel. units]	1.00±0.13	1.17±0.09	1.07±0.11
LDHA [rel. units]	1.00±0.08	1.19±0.14	2.41±0.25 *§
PFKL [rel. units]	1.00±0.05	1.25±0.15	1.94±0.21 *§
PGK1 [rel. units]	1.00±0.04	1.29±0.12 *	1.57±0.09 *§
PHD2 [rel. units]	1.00±0.03	1.18±0.06	1.50±0.13 *§
PKM [rel. units]	1.00±0.03	1.27±0.25 *	1.38±0.11 *

*, p<0.05 versus sham; § p<0.05 versus 2K1C; sham, control sham operation; 2K1C, 2-kidney-1-clip hypertensive rats; ICA, 2-(1-chloro-4- hydroxyisoquinoline- 3-carboxamido) acetate; EPO, erythropoietin; HO, heme oxigenase; HIG, HIF-inducible gene; VEGF, vascular endothelial growth factor; IGFBP, insulin like growth factor binding protein; ADM, adrenomedullin; ET, endothelin; iNOS, inducible NO synthase; Aldo, aldolase; BNIP-3, BCL2 interacting protein 3; CA-9, Carbonic anhydrase 9; CCND1, Cyclin D1; LDHA, Lactate dehydrogenase A; PFKL, Phosphofructokinase liver type; PGK1,

Phosphoglycerate kinase 1; PHD2, Prolyl hydroxylase domain containing protein 2; PKM, Pyruvate kinase M1/M2.

Supplemental Table 4: HIF Targets in left ventricle 4 hours after administration of ICA

**mRNA expression**	**sham**	**2K1C + placebo**	**2K1C + ICA**
HO-1 [rel. units]	1.00±0.48	1.55±0.59	1.07±0.48
HIG-2 [rel. units]	1.00±0.11	1.37±0.21	1,27±0.07
VEGFA [rel. units]	1.00±0.23	1.47±0.10	1,63±0.17 *
ET-1 [rel. units]	1.00±0.17	2.54±0.57 *	1.89±0.31
IGFBP-3 [rel. units]	1.00±0.31	0.61±0.06	0.75±0.06
ADM [rel. units]	1.00±0.09	1.90±0.26 *	1.65±0.12 *
iNOS [rel. units]	1.00±0.22	0.90±0.12	1.68±0.29 *§
AldoA [rel. units]	1.00±0.13	1.40±0.06 *	1.46±0.07 *
AldoC [rel. units]	1.00±0.11	1.96±0.16 *	1.97±0.11 *
BNIP-3 [rel. units]	1.00±0.11	1.67±0.32	1.49±0.19
CA-9 [rel. units]	1.00±0.25	0.66±0.14	0.89±0.25
CCND1 [rel. units]	1.00±0.09	1.41±0.11 *	1.63±0.14 *
LDHA [rel. units]	1.00±0.12	1.23±0.05	1.20±0.11
PFKL [rel. units]	1.00±0.09	1.46±0.07 *	1.59±0.11 *
PGK1 [rel. units]	1.00±0.11	1.33±0.05 *	1.46±0.08 *
PHD2 [rel. units]	1.00±0.11	1.34±0.06 *	1.47±0.06 *
PKM [rel. units]	1.00±0.13	1.71±0.07 *	1.80±0.08 *

*, p<0.05 versus sham; § p<0.05 versus 2K1C; sham, control sham operation; 2K1C, 2-kidney-1-clip hypertensive rats; ICA, 2-(1-chloro-4- hydroxyisoquinoline- 3-carboxamido) acetate; HO, heme oxigenase; HIG, HIF-inducible gene; VEGF, vascular endothelial growth factor; IGFBP, insulin like growth factor binding protein; ADM, adrenomedullin; ET, endothelin; iNOS, inducible NO synthase; Aldo, aldolase; BNIP-3, BCL2 interacting protein 3; CA-9, Carbonic anhydrase 9; CCND1, Cyclin D1; LDHA, Lactate dehydrogenase A; PFKL, Phosphofructokinase liver type; PGK1, Phosphoglycerate kinase 1; PHD2, Prolyl hydroxylase domain containing protein 2; PKM, Pyruvate kinase M1/M2.

Supplemental Table 5: Right renal expression of markers of tissue fibrosis and inflammation

**mRNA expression [rel. units]**	**sham**	**2K1C + placebo**	**2K1C + ICA**
Collagen I	1.00±0.09	4.35±0.96 *	3.74±0.82
Fibronectin	1.00±0.09	4.57±0.69 *	3.70±0.52 *
TGFβ1	1.00±0.05	2.86±0.30 *	2.30±0.27 *
TNFα	1.00±0.07	1.67±0.17	1.90±0.40
IL-1b	1.00±0.12	2.27±0.27	4.43±1.82
IL-6	1.00±0.13	16.83±4.05 *	18.27±5.00 *
IL-11	1.00±0.05	20.15±3.33 *	19.02±3.65 *
MCP-1	1.00±0.11	4.27±0.66 *	3.80±0.64 *
CCL-5	1.00±0.09	1.22±0.12	1.13±0.20
CCL-7	1.00±0.10	4.32±0.71 *	3.34±0.60 *
CXCL-6	1.00±0.26	11.17±2.33 *	11.43±2.78 *
Osteopontin	1.00±0.05	56.55±7.52 *	67.18±9.19 *
ICAM-1	1.00±0.03	3.26±0.38 *	3.99±0.60 *
VCAM	1.00±0.08	1.44±0.12 *	1.36±0.15

*, $p<0.05$ versus sham; sham, control sham operation; 2K1C, 2-kidney-1-clip hypertensive rats; ICA, 2-(1-chloro-4- hydroxyisoquinoline- 3-carboxamido) acetate.

Supplemental Table 6: Left ventricular expression of markers of tissue fibrosis and inflammation

mRNA expression [rel. units]	sham	2K1C + placebo	2K1C + ICA
Collagen I	1.00±0.05	2.01±0.46	1.94±0.32
Fibronectin	1.00±0.09	4.27±1.02 *	4.21±0.66 *
TGFβ1	1.00±0.08	1.35±0.15	1.63±0.13 *
TNFα	1.00±0.16	1.33±0.11	1.58±0.14 *
IL-1b	1.00±0.14	1.77±0.24	3.88±1.36 *
IL-6	1.00±0.08	7.17±1,78	47.57±25.80
IL-11	1.00±0.16	1.51±0.19	4.55±2.46
MCP-1	1.00±0.21	3.78±1.24	6.78±3.67
CCL-5	1.00±0.16	0.51±0.06 *	0.51±0.08 *
CCL-7	1.00±0.16	4.03±1.40	8.57±4.45
Osteopontin	1.00±0.19	20.37±10.19	16.08±3.14
ICAM-1	1.00±0.09	1.48±0.15	2.52±0.62 *
VCAM	1.00±0.15	1.38±0.14	1.36±0.30

*, $p<0.05$ versus sham; sham, control sham operation; 2K1C, 2-kidney-1-clip hypertensive rats; ICA, 2-(1-chloro-4- hydroxyisoquinoline- 3-carboxamido) acetate.

Supplemental Table 7: Right renal expression of angiogenetic markers

**mRNA expression [rel. units]**	**sham**	**2K1C + placebo**	**2K1C + ICA**
EPO	1.00±0.12	3.30±1.19	19.70±13.30
VEGF-A	1.00±0.04	1.15±0.09	1.04±0.08
VEGF-B	1.00±0.07	1.08±0.10	0.88±0.06
VEGF-C	1.00±0.09	1.79±0.15 *	1.44±0.13
VEGF-D	1.00±0.09	1.51±0.13 *	1.20±0.18
VEGF-R1	1.00±0.05	1.09±0.09	1.02±0.09
VEGF-R2	1.00±0.07	0.91±0.07	0.74±0.06 *
VEGF-R3	1.00±0.08	2.39±0.23 *	2.02±0.29 *
AP-1	1.00±0.12	1.02±0.09	0.76±0.08 §
AP-2	1.00±0.10	1.94±0.16 *	2.06±0.30 *
Tie-1	1.00±0.08	1.15±0.08	0.85±0.08 §
Tie-2	1.00±0.08	1.20±0.07	1.04±0.08
PlGF	1.00±0.09	2.42±0.29 *	2.43±0.40 *

*, p<0.05 versus sham; §, p<0.05 versus 2K1C + placebo; sham, control sham operation; 2K1C, 2-kidney-1-clip hypertensive rats; ICA, 2-(1-chloro-4- hydroxyisoquinoline- 3-carboxamido) acetate; VEGF, vascular endothelial growth factor; VEGF-R, vascular endothelial growth factor receptor; AP, angiopoietin; Tie, angiopoietin receptor; PlGF, placental growth factor.

Supplemental Table 8: Left ventricular expression of angiogenetic markers

**mRNA expression [rel. units]**	**sham**	**2K1C + placebo**	**2K1C + ICA**
VEGF-A	1.00±0.17	0.94±0.07	0.71±0.07 *§
VEGF-B	1.00±0.08	0.71±0.05 *	0.58±0.07 *
VEGF-C	1.00±0.12	1.03±0.06	0.86±0.07
VEGF-D	1.00±0.12	1.82±0.20 *	2.18±0.31 *
VEGF-R1	1.00±0.13	1.14±0.08	1.11±0.08
VEGF-R2	1.00±0.11	1.12±0.10	0.86±0.08
VEGF-R3	1.00±0.12	0.99±0.08	0.87±0.09
AP-1	1.00±0.08	0.47±0.14 *	0.43±0.06 *
AP-2	1.00±0.07	0.81±0.06	0.88±0.08
Tie-1	1.00±0.10	0.83±0.07	0.60±0.06 *§
Tie-2	1.00±0.07	1.04±0.07	0.86±0.07
PlGF	1.00±0.08	0.72±0.09 *	0.81±0.07

*, p<0.05 versus sham; §, p<0.05 versus 2K1C + placebo; sham, control sham operation; 2K1C, 2-kidney-1-clip hypertensive rats; ICA, 2-(1-chloro-4- hydroxyisoquinoline- 3-carboxamido) acetate. VEGF, vascular endothelial growth factor; VEGF-R, vascular endothelial growth factor receptor; AP, angiopoietin; Tie, angiopoietin receptor; PlGF, placental growth factor
